# Improvements on Restricted Insecticide Application Protocol for Control of Human and Animal African Trypanosomiasis in Eastern Uganda

**DOI:** 10.1371/journal.pntd.0003284

**Published:** 2014-10-30

**Authors:** Dennis Muhanguzi, Kim Picozzi, Jan Hatendorf, Michael Thrusfield, Susan Christina Welburn, John David Kabasa, Charles Waiswa

**Affiliations:** 1 Department of Biomolecular and Biolaboratory Sciences, College of Veterinary Medicine Animal Resources and Biosecurity, Makerere University, Kampala, Uganda; 2 Division of Pathway Medicine, Centre for Infectious Diseases, School of Biomedical Sciences, College of Medicine and Veterinary Medicine, The University of Edinburgh, Edinburgh, United Kingdom; 3 Department of Public Health and Epidemiology, Swiss Tropical Institute, Basel, Switzerland; 4 University of Basel, Basel, Switzerland; 5 Royal (Dick) School of Veterinary Studies, The University of Edinburgh, Edinburgh, United Kingdom; International Centre of Insect Physiology and Ecology, Kenya

## Abstract

**Background:**

African trypanosomes constrain livestock and human health in Sub-Saharan Africa, and aggravate poverty and hunger of these otherwise largely livestock-keeping communities. To solve this, there is need to develop and use effective and cheap tsetse control methods. To this end, we aimed at determining the smallest proportion of a cattle herd that needs to be sprayed on the legs, bellies and ears (RAP) for effective Human and Animal African Trypanosomiasis (HAT/AAT) control.

**Methodology/Principal finding:**

Cattle in 20 villages were ear-tagged and injected with two doses of diminazene diaceturate (DA) forty days apart, and randomly allocated to one of five treatment regimens namely; no treatment, 25%, 50%, 75% monthly RAP and every 3 month Albendazole drench. Cattle trypanosome re-infection rate was determined by molecular techniques. ArcMap V10.3 was used to map apparent tsetse density (FTD) from trap catches. The effect of graded RAP on incidence risk ratios and trypanosome prevalence was determined using Poisson and logistic random effect models in R and STATA V12.1 respectively. Incidence was estimated at 9.8/100 years in RAP regimens, significantly lower compared to 25.7/100 years in the non-RAP regimens (incidence rate ratio: 0.37; 95% CI: 0.22–0.65; P<0.001). Likewise, trypanosome prevalence after one year of follow up was significantly lower in RAP animals than in non-RAP animals (4% vs 15%, OR: 0.20, 95% CI: 0.08–0.44; P<0.001). Contrary to our expectation, level of protection did not increase with increasing proportion of animals treated.

**Conclusions/significance:**

Reduction in RAP coverage did not significantly affect efficacy of treatment. This is envisaged to improve RAP adaptability to low income livestock keepers but needs further evaluation in different tsetse challenge, HAT/AAT transmission rates and management systems before adopting it for routine tsetse control programs.

## Author Summary

Poverty, hunger and human ill-health aggravated by trypanosomiasis in Sub-Saharan Africa can only be reduced by developing and using cheap and effective tsetse control methods. To further reduce the cost of tsetse control by restricting insecticides to the legs, belly and ears (RAP) we set out to determine the lowest RAP coverage that can effectively control tsetse. Cattle in 20 south-eastern Uganda villages were randomly allocated to 5 treatment groups, ear-tagged for ease of follow-up and treated twice forty days apart with a trypanocide at the beginning of the trial. Cattle in regimens 2–4 received monthly graded RAP (25%, 50% and 75% of village herd respectively), while those in regimens 1 and 5 received no more treatment and deworming once every three months respectively. Molecular techniques were used to check for trypanosome infections, while tsetse apparent density was determined by traps at 161 locations in the district. About 25% RAP coverage was effective at controlling *T. brucei s.l.* while 50–75% RAP coverage would need to be used for effective *T.vivax* and *T.congolense* nagana control. Use of RAP at lower herd coverage is envisaged to reduce its cost, damage to the environment and improve its uptake in resource poor communities.

## Introduction

African trypanosomes transmitted by tsetse flies (Diptera: Glossinidae) pose one of the biggest constraints to animal and human health and livestock-crop integration in Sub-Saharan Africa [1], [2]. They cause a debilitating disease in domestic animals (nagana) and humans (sleeping sickness) [3]–[5]. In the south-eastern part of Uganda, cattle are the main reservoir of *Trypanosoma brucei rhodesiense* the causative agent of the acute form of African human trypanosomiasis (HAT) [6]–[9]. The chronic form of the disease caused by *T. b. gambiense*, whose main reservoir is yet unknown, exists in West Nile Districts of Uganda extending to most parts of South–Sudan [10], [11]. However, active case detection and management have been shown to be effective in *T.b. gambiense* control indicating that humans are very important in maintaining disease transmission [12]–[14].

The distance between the two forms of HAT has been decreasing threatening a merger as a result of massive cattle restocking in south-eastern Uganda following 20 years of unrest in this region [10], [15], [16]. This merger has recently been temporarily halted by the Stamp-out sleeping sickness (SOS) program-led preventive chemotherapy and pyrethroid insecticide spraying of about 0.5 million cattle [17], [18]. However, this halt remains temporary unless control efforts are sustained [18], [19].

The above notwithstanding, The World Bank estimates that about 25% of the population in Sub-Saharan Africa and Uganda in particular, subsists on less than US $ 1.25 per day. This poverty level is compounded by food insecurity that affects over 34% of the population [20], [21] and ill-health caused by HAT in addition to other endemic human diseases in this region. However, the majority of the poor people in Ugandan communities afflicted by HAT own cattle [22], [23] whose production is also constrained by AAT. This implies that improving livestock production has potential to reduce poverty and improve food security [1], [2], [20], [24]. Before this can be achieved, there is need to put in place effective and sustainable HAT/AAT control methods. Such control methods to be effective and sustainable in small holder crop-livestock production systems need to be commensurate to inelastic budgets of small holder livestock keepers. In addition, they need to be environmentally benign and target more than one of the endemic diseases that are known to occur in these areas.

Previously, restricting pyrethroid insecticides to the belly and legs had proved cheap, environmentally benign and unequivocally effective on tsetse and trypanosomiasis control compared to other control methods [25]–[27]. It has also been suggested that RAP is unlikely to disrupt endemic stability to tick-borne diseases (TBDs); an epidemiological equilibrium that is known to maintain a large population of cattle protected against TBDs [27]. However, it had been suggested that RAP needs to be optimized in the field setup so as to further reduce its cost and make it commensurate to inelastic budgets of small holder livestock keepers [27]. To this end, a cluster randomized trial was carried out to determine the smallest proportion of a village herd that needs to be sprayed by restricting pyrethroid insecticides to the bellies, legs and ears of cattle and effectively control HAT/AAT. To achieve this, bovine trypanosome prevalences were determined by molecular techniques before and after spraying (by RAP) 0%, 25%, 50%, 75% of village cattle herds in 20 villages in Tororo, district; eastern Uganda. RAP was initially developed basing on a body of research that indicated that tsetse land and feed mostly on legs, bellies and ears of the larger compared to smaller/younger cattle [27]–[29]. Restricting insecticides to the legs, bellies and ears reduces the amount of the insecticide by 5 fold; reducing on the cost of application and environmental effects to the dung fauna that break down dung into manure [25], [30]. It is upon this background that we sought to further optimize RAP.

## Materials and Methods

### Study area; study village selection and allocation to treatment regimens

This study was carried out in Tororo district, south-eastern Uganda for 18 months between June 2012–December 2013. *Glossina fuscipes fuscipes* and *Glossina pallidipes* are the main tsetse fly vectors of trypanosomiasis in this area [9], [31]. The location, livestock production systems, climate and vegetation of Tororo district have been described elsewhere [32], [33]. The 20 intervention villages were selected from 57 villages of a larger survey of trypanosome (D Muhanguzi; unpublished) and *T.parva* [33] prevalence in Tororo district by molecular techniques. Fifty seven villages were screened for eligibility and data collected on basic socio-demographics and trypanosome prevalence by molecular techniques. Twenty-seven villages fulfilled the eligibility criteria of i) a cattle population of > = 50 and ii) a trypanosome prevalence of > = 15%. A village cattle population of 50 was used so as to make sure that cattle population is large enough not to be depleted in 18 months of follow-up. Baseline trypanosome prevalence of 15% was used for village inclusion in order to provide a wide enough range to be able to measure the effect of graded RAP on trypanosome prevalence. In order to select 20 villages, 100 unique allocation sequences were generated which fulfilled the condition of a minimum distance of 2 km between neighbouring villages. This was to minimize contamination effects from different intervention arms. Finally, one allocation sequence was selected randomly.

### Description of field cattle treatments

Each of the 20 study villages was randomized to one of five different treatments. All cattle in 20 study villages were ear tagged for ease of identification at follow-up. They were then treated with a short acting diminazene diaceturate (DA) containing cyanocobalamin (vitamin B12) and hydroxocobalamin (Vitamin B12a) (Veriben B12; Ceva santé animale, France) at the beginning of the trial. Another DA dose was administered 40 days later to all cattle in the 20 study villages to clean them of residual trypanosome infections and be able to monitor the rate at which re-infection took place. Diminazene diaceturate was administered at a dose of 0.01 g/kg live body weight (bwt) by deep intramuscular injection. In order to assess, herd structure (age, sex, breed, exits/entries) at each sampling time, livestock-keepers, their household particulars (village, parish, county) and cattle demographics were entered on a herd structure register at the time of introduction into the intervention. This register was updated once three monthly for 15 months. In regimens 2–4; different proportions (25%, 50% and 75%) of the village herd were sprayed once every 28 days in what is referred to here as graded RAP. This was to determine the effect of spraying different proportions of a village cattle herd on the rate of transmission of different trypanosomes. An emulsifiable deltamethrin concentrate (Vectocid, Ceva Interchem, Tunis) spray was applied in the recommended concentration of 1; 1000 (Vectocid to water parts) on legs, belly and ears as previously described [27]. The first 25%, 50% and 75% of all the registered cattle to be presented in the respective RAP regimens were spayed at each of the monthly spraying. Cattle in regimen 5 were in addition given an Albendazole 10% drench at a dose rate of 0.008 g/kg bwt once after three months. This was to create a replica non-RAP regimen where a non tsetse and-trypanosomiasis effective treatment was administered as an incentive for farmers to present cattle for trypanosome testing for 18 months. This was introduced in the design of this study in order to reduce the risk of excessive losses to follow-up in Regimen 1. As such, regimens 1 and 5 were planned control regimens for RAP regimens 2–4. Blood samples were taken 14 days post the last Veriben B12 injection and repeated once three monthly for 18 months of the trial in order to monitor the rate of re-infection with different trypanosomes. For ethical reasons, all cattle in the non-RAP villages were administered with Veriben B12 injections at the end of the trial since they were at a higher risk of infection during the trial.

### Cattle blood sample collection

About 125 µl of blood were collected from the middle ear vein and applied onto designated sample area of the classic Whatman FTA cards (Whatman Bioscience, Cambridge, UK) avoiding cross contamination [34], [35]. Blood samples were then allowed to air-dry, labelled with cattle ear tag numbers, treatment regimen, sampling number, village name, parish, sub County, County and date of collection. They were packed in foil pouches with a silica gel desiccant (Sigma Aldrich, Co., Life sciences, USA) prior to shipping to the University of Edinburgh, UK for analysis.

### DNA extraction

DNA was extracted and eluted in Chelex-100 resin (Sigma Aldrich, Co., Life sciences, USA) from five 3 mm FTA sample discs according to a previously described protocol [35], [36]. Eluted DNA samples were kept at −20°C for long-term PCR analyses or 4°C if they were to be analysed within a few days after extraction.

### Trypanosome detection

Eluted DNA samples were screened for different trypanosome species using a single pair of primers (CR and BR) and thermo cycling conditions as previously described [37]. The ITS1- PCR was done in 25 µl reaction volume; 20 µl of which were the PCR master-mix and either 5 µl of the test sample or negative control eluate or positive control DNA. The master-mix was made of 10×-reaction buffer (670 mM Tris-HCl pH 8.8, 166 µM (NH4)_2_SO_4_, 4.5% Triton X-100, 2 mg/ml gelatin) (Fisher Biotech), 1.0 mM MgCl_2_, 200 µM of each dNTP, 5 µM each of the CF and BR primers, 0.5 U of *Taq* DNA polymerase (Fisher Biotech) and 15.2 µl RNase-free (molecular grade) water.

To determine which samples were infected with either *T. brucei* or *T. b. rhodesiense*, multiplex PCR [38] was carried out on each of the samples from which a 450 bp fragment was detected on ITS1-PCR. Multiplex PCR was done in 25 µl reactions using primers and conditions as previously described [38].

In order to determine the commonest *T.congolense* genotype circulating in Tororo district, all samples from which a ≥600 bp fragment was amplified on ITS1-PCR were initially tested for *T.congolense savannah* using a single pair of primers (TCS1 & TCS2) and thermo cycling conditions as previously described [39]. All samples that were positive for *T.congolense* DNA on ITS1-PCR were positive for *T.congolense savannah*. For this reason, no more *T.congolense* genotype-specific (Kilifi, Tsavo, forest) PCRs were performed although a few co-infections with different *T.congolense* genotypes could have been possible. The PCR was done in 25 µl reaction volume; 20 µl of which were the PCR master-mix and either 5 µl of the test sample or negative control eluate or positive control DNA. The master-mix was made of 10×-reaction buffer (670 mM Tris-HCl pH 8.8, 166 µM(NH4)_2_SO_4_, 4.5% Triton X-100, 2 mg/ml gelatin) (Fisher Biotech),, 4.5% Triton X-100, 2 mg/ml gelatin) (Fisher Biotech), 0.75 mM MgCl_2_, 200 µM of each dNTP, 12.5 µM each of the TCS1 & TCS2 primers, 1 U of *Taq* DNA polymerase (Fisher Biotech) and 13.05 µl of RNase-free water.

PCR products for the three sets of PCRs were electrophoresed in 1.5% agarose (Bio Tolls Inc. Japan), stained in GelRed (Biotium, Inc., USA) and visualised on a UV transilluminator for fragment size determination.

### Determination of apparent tsetse density (FTD)

Pyramidal traps [40] were set in 161 locations by Tororo District Entomology Department between June and September 2012. Individual tsetse trap catches were used to determine pre-intervention FTD. About 12 traps were set per Km^2^ to cover at least a square km (km^2^) of each sub county. Tsetse fly catches were monitored, emptied and species and sex determined after every 24 hours. Apparent tsetse density was determined as the number of tsetse flies per trap per day.

### Statistical analysis

The primary analysis investigated the impact of RAP on the incidence risk ratios of any trypanosome infection using generalized linear mixed models with a Poisson distribution and a logarithmic link function. To account for correlation within clusters, villages were included as gamma distributed random effects. The logarithm of the time under observation, i.e. the time period between the first and last time an individual animal was sampled, was included as offset variable. To assess the intervention effect over time, prevalences after 12 and 18 months of follow up were compared using mixed models with binary outcome and logit link function. Additional analyses at other sampling points are provided in supporting information S1. The original idea of modelling the proportion of animals treated with RAP as a dose response relationship was abandoned because incidence did not decrease with increasing proportion of treated animals. Therefore, the results for the different treatment regimens compared to the control regimens are presented.

Apparent tsetse density was determined as the number of tsetse captured per trap per day. To determine the spatial distribution of tsetse flies (*G.pallidipes* and *G.fuscipes*) in Tororo district an FTD map was generated using the Inverse Weighing Distance Extension (IDW) [41] of ArcMap 10.3 of 161 individual trap catches. Interpolation was done at two spatial resolutions (grid cell sizes of 1 km^2^ and 25 km^2^) and raster values were extracted for each village at each spatial resolution. A default exponent value of 2 was chosen. Although there was little evidence of spatial autocorrelation (Moran's I = −0.11, −0.08, −0.10; all P >0.2, for baseline trypanosome prevalence and FTDs at 1 km^2^ and 25 km^2^ resolution, respectively. The association between FTD and trypanosome prevalence was adjusted for potential spatial dependence. We used a generalized least squares model with a Gaussian spatial correlation structure to quantify the effect. Statistical analyses were performed using R v 3.0.2 (packages ‘lme4’, ‘nlme’ and ‘ape’) except Poisson random effect models which were performed in STATA v 12.1.

### Ethical clearance

This study was reviewed and approved by the Makerere University College of Veterinary Medicine Animal Resources and Biosecurity (COVAB) research and ethics committee for consistency to animal use and care. Upon approval (number VAB/REC/10/105) the COVAB research and ethics committee forwarded it to the Uganda National Council for Science and Technology (UNCST) and it was further approved and registered under registration number HS1336.

## Results

### Study flow

**Figure 1:**
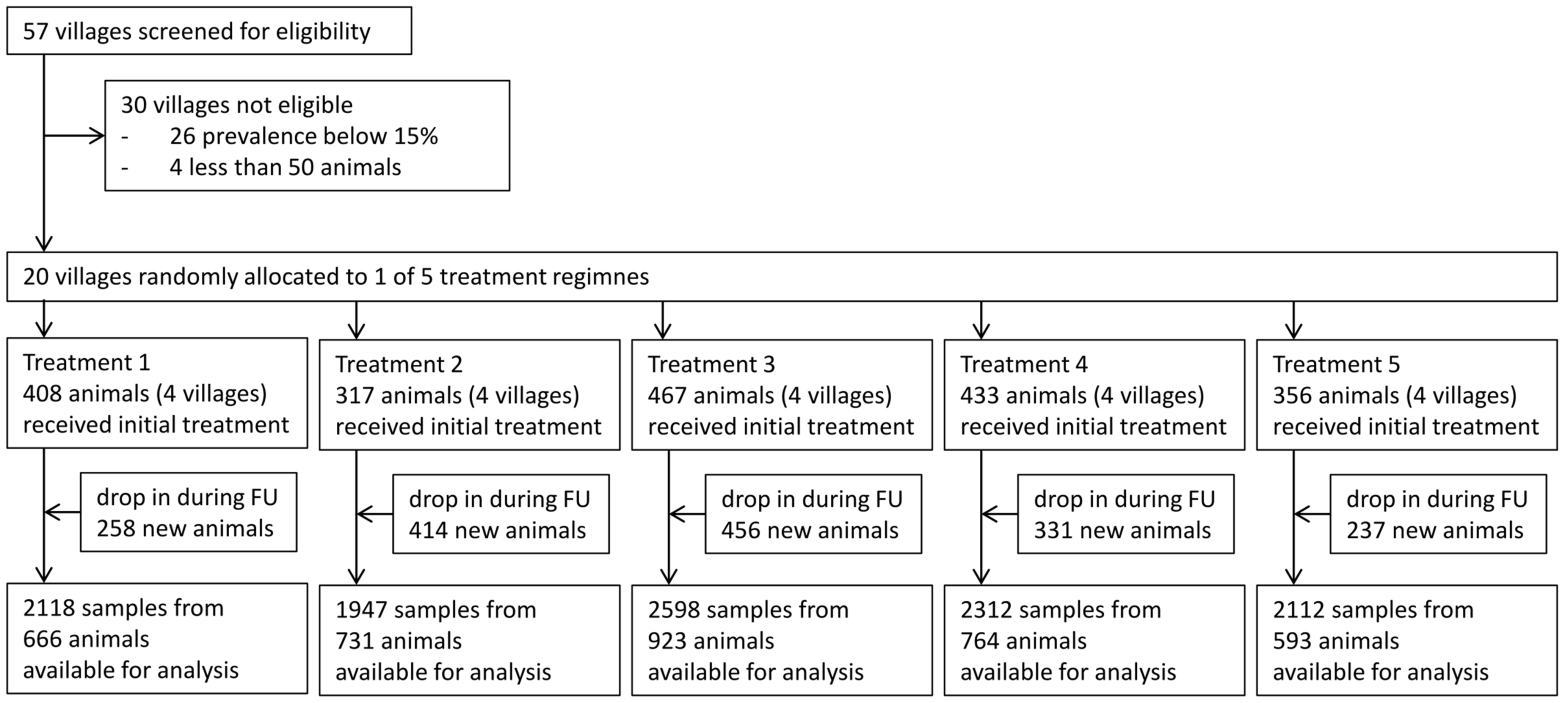
Study flow. Regimen 1: Diminazene diaceturate injections (DA); (0.01 g/kg body weight) forty days apart at the beginning of the trial. Regimen 2: DA and 25% RAP. Regimen 3: DA and 50% RAP. Regimen 4: DA and 75% RAP. Regimen 5: DA and Albendazole 10% drench (8 mg/kg body weight)-3 monthly for 18 months. Median time of follow up-FU (time difference between first and last sampling of individual animals) was 12 months in each of the 5 treatment groups.

Over all seven time points, eleven thousand blood samples (11,087) were collected from 3,677 cattle. One thousand nine hundred eighty one cattle (54%) were sampled 14 days post the second Veriben B12 injections and examined to determine trypanosome residual infections. Almost half the investigated animals (46%) were newly introduced into the herd during the 18 months of follow up (Figure 1).

### Demographic characteristics 2 weeks after the initial treatments

Pre-intervention trypanosome prevalence ranged from 20–27% in different regimens. The Boran and African short horn zebu hybrid was the most predominant cattle breed (98%) and well balanced among the five treatment groups (range 93%–100%). Treatment groups were slightly imbalanced with respect to age and sex composition. Roughly half of the animals were above 3 years of age (Table 1).

**Table 1:** Baseline characteristics of the study population (2 weeks after initial treatment).

Prevalence indicators	Regimen					RAP status	
	1	2	3	4	5	no RAP (1 &5)	RAP (2–4)
**A) Infection status 9 months before treatment and 2 weeks after initial treatment**							
Cattle (n)	408	317	467	433	356	764	1217
Villages (n)	4	4	4	4	4	8	12
Prevalence before treatment [%]* ¶	27	27	20	23	22	24	23
Prevalence after treatment [%]¶	0	1	2	1	3	2	1
**B) Demographic characteristics**							
**Population attributes**	**n (% Within group)**						
i) Sex [n (%)]							
Male	175 (43%)	109 (34%)	165 (35%)	176 (41%)	144 (40%)	319 (42%)	451 (37%)
Female	192 (47%)	193 (60%)	269 (58%)	226 (52%)	197 (55%)	56 (7%)	81 (7%)
Neutered	41 (10%)	18 (6%)	33 (7%)	30 (7%)	15 (5%)	389 (51%)	685 (56%)
ii) Breed [n (%)]							
Boran×African short horn Zebu (Nkedi);	394 (97%)	295 (93%)	450 (96%)	424 (98%)	355(100%)	749 (98%)	1169 (96%)
Boran×Holstein Friesian	8 (2%)	21 (7%)	0 (0%)	3 (1%)	1 (0%)	9 (1%)	24 (2%)
African short horn Zebu (Nkedi)	6 (1%)	1 (0%)	17 (4%)	6 (1%)	0 (0%)	6 (1%)	24 (2%)
iii) Age [n (%)]							
0–1 years	47 (12%)	39 (12%)	35 (7%)	72 (17%)	70 (20%)	117 (15%)	146 (12%)
1.1–3 years	189 (46%)	153 (48%)	188 (40%)	151 (35%)	119 (33%)	308 (40%)	492 (40%)
>3.1 years	172 (42%)	125 (39%)	244 (52%)	210 (48%)	167 (47%)	339 (44%)	579 (48%)

* Determined 9 months before treatment (n = 321, 430, 572, 576, 509),

¶ Infected with either *T.vivax. T.b.brucei, T.b.rhodesiense* and *T.c. savannah*.

### Prevalence of different trypanosome species by regimen and time

**Figure 2:**
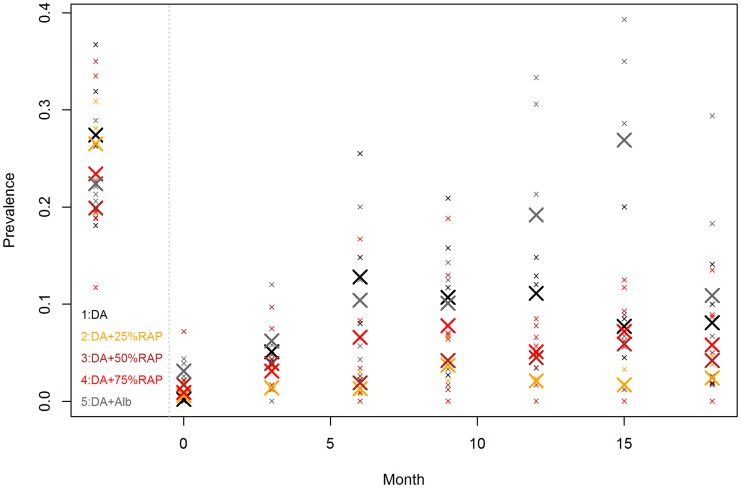
Trypanosome prevalence by time in different regimens. Small symbols represent the prevalence in the investigated villages. The large symbols represent the mean value of the 4 prevalence estimates in each regime. Animals infected with either *T.vivax*. *T.b.brucei, T.b. rhodesiense, T.c.savannah* are considered infected. The data left to the dotted vertical line denote the baseline estimates determined about 9 months before treatment.

**Figure 3:**
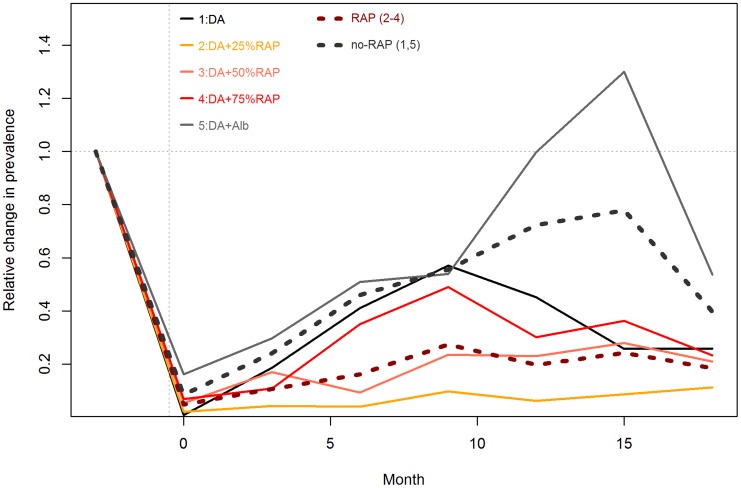
Relative Change in trypanosome prevalence by Regimen 1–5. Lines represent the relative changes from the baseline prevalences, presented are the means from the 4 village estimates. The dotted lines represent average prevalence in RAP (2–9; brown) and non- RAP (1&5; black) regimens respectively.

Fourteen days post the second dose of diminazene diaceturate (denoted as time 0), trypanosome prevalences generally increased in all regimens up to month 6 when they started decreasing (Regimen 2, 3 and 4) over time. In regimens 1 and 5 trypanosome prevalences increased up to about 12 and 15 months respectively and started decreasing thereafter. The slope of curves representing trypanosome prevalences over time in different regimens is in increasing order of Regimen 2<3<4<1<5 (Figures 2& 3). *T.vivax* was the most predominant species detected in any regimen while *T. brucei s.l.* was the least predominant species detected over the study period (Table 2).

**Table 2:** Overall prevalence [%] of different trypanosome species in different treatment regimens over an 18 months follow-up period.

Regimen	Month	n	*T.vivax*	*T.b.brucei*	*T.c.savannah*	Any Tryps	Number (n*)	Any Tryps*
1	0	408	0.2	0.0	0.0	0.2	na	na
2		317	0.6	0.0	0.0	0.6	na	na
3		467	1.5	0.2	0.0	1.7	na	na
4		433	0.9	0.0	0.0	0.9	na	na
5		356	3.1	0.0	0.0	3.1	na	na
1	3	273	4.0	0.4	1.1	5.1	268	4.9
2		294	1.0	0.0	0.3	1.4	294	1.4
3		415	3.6	0.0	0.2	3.9	414	3.9
4		355	2.8	0.0	0.3	3.1	338	3
5		355	5.9	0.0	0.3	6.2	353	5.9
1	6	359	8.9	1.4	2.5	12.8	316	13.3
2		308	1.0	0.3	0.0	1.3	264	1.1
3		375	1.9	0.0	0.0	1.9	326	2.1
4		361	5.0	0.8	1.1	6.6	309	5.5
5		316	10.1	0.6	0.3	10.4	263	11
1	9	307	9.1	0.3	1.6	10.7	257	11.7
2		299	2.7	0.3[1]	0.7	3.7	231	3.9
3		383	3.9	0.3	0.0	4.2	259	4.2
4		320	6.9	0.3	0.6	7.8	226	7.5
5		356	10.1	0.6	0.0	10.1	236	11
1	12	404	8.9	1.0	1.2	11.1	331	12.4
2		285	1.8	0.4	0.0	2.1	186	3.2
3		426	4.5	0.0	0.0	4.5	297	4.4
4		353	4.5	0.0	0.6	5.1	230	4.3
5		339	18.0	2.1	1.2	19.2	248	21
1	15	169	4.1	1.2	2.4	7.7	150	8.7
2		234	1.7	0.0	0.0	1.7	124	0.8
3		270	5.9	0.0	0.0	5.9	148	5.4
4		264	6.1	0.0	1.1	7.2	136	8.1
5		197	24.9	3.0	2.0	26.9	134	26.1
1	18	198	4.5	0.5	3.0	8.1	173	7.5
2		210	2.4	0.0	0.0	2.4	90	3.3
3		262	3.8	0.8	0.0	4.2	117	6.8
4		226	4.4	0.4	1.3	5.8	128	6.2
5		193	9.3	0.0	2.1	10.9	137	10.2

Any Tryps = Infected with any trypanosome species namely; *T.vivax*. *T.b.brucei, T.b.rhodesiense*, *T.congolense* (*savannah*); na; not applicable.

[1] ; one sample positive for SRA gene (*T.b. rhodesiense*).

* Only animals which received the initial DA are included.

### Incidences and point prevalence of different trypanosomes in RAP and Non-RAP regimens

**Figure 4:**
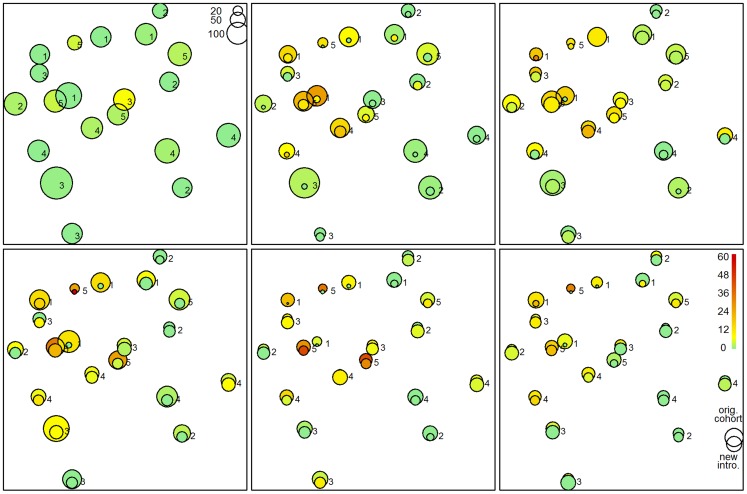
Herd (village) level effects on trypanosome prevalences with time and regimen. Spatial distribution (any trypanosome) over an 18 months period. Comparison “original cohort” (upper circle: animals which got initial DA treatment) and new animals (lower circle: tagged first time during follow up). The colours represent the prevalences. The circle area is proportional to the number of sampled animals.

At the end of follow-up, we observed an incidence of 9.8 per 100 animal years in the RAP regimens which was significantly lower compared to the 25.7 in the non RAP regimens (incidence rate ratio: 0.37; 95% CI: 0.22–0.65; P<0.001). Likewise, trypanosome prevalence after one year of follow up was 15% in the non-RAP regimens compared to 4% in the RAP animals (OR: 0.20, 95% CI: 0.08–0.44; P<0.001). The effect was lower but statistically significant after 18 months of follow up (9% vs 4%; OR: 0.38; 95% CI: 0.14–0.93; P = 0.03). Adjustment for sex, age category, FTD at 1 km^2^ spatial aggregation (FTD-1000 m) and for animal treatment at baseline did not noteworthy change the estimates (Table 3). There was some indication that FTD-1000 m had an impact on the treatment effect, but the association was only statistically significant in one of the 3 models. Newly introduced animals had slightly lower risk but it was only marginally significant. Of note, newly introduced cattle were generally younger (median age 2.3 years compared to 4.0 years). As such, trypanosome infections were persistently higher in isolated villages in central, northern and western parts of Tororo District especially in Kirewa, Nagongera and Paya sub counties (Figure 4). Details of the models on the other sampling dates as well as time×treatment interaction are provided in supporting information S1.

**Table 3:** Incidence and prevalence of different bovine trypanosomes during 18 months follow-up period in RAP and non RAP regimens.

a) Incidence				
Descriptive statistics				
	Sampled (n)	Follow-up years	Positive (n)	Incidence per 100 years
No RAP	1205	1246	320	25.7
RAP	2250	2164	211	9.8
**Inferential statistics**				
Model	variable	IRR	95% CI	P
unadjusted	RAP	0.37	0.22–0.65	<0.001
adjusted	RAP	0.36	0.21–0.62	<0.001
	age 1–3 vs >3 yrs	1.16	0.97–1.4	0.11
	age <1 vs >3 yrs	0.97	0.73–1.28	0.82
	sex male	0.93	0.85–1.02	0.14
	FTD-1000 m	1.12	0.92–1.38	0.26
	new animal	0.86	0.69–1.06	0.16

b) Prevalence at month 12				
Descriptive statistics				
	Sampled (n)	Positive (n)	Positive (%)	
no RAP	743	110	15	
RAP	1064	43	4	
**Inferential statistics**				
Model	variable	OR	95% CI	P
unadjusted	RAP	0.20	0.08–0.44	<0.001
adjusted	RAP	0.22	0.10–0.48	0.0001
	age 1–3 vs >3 yrs	0.41	0.05–3.15	0.39
	age <1 vs >3 yrs	0.95	0.66–1.36	0.79
	sex male	0.99	0.72–1.35	0.97
	FTD-1000 m	1.58	1.11–2.26	0.01
	new animal	0.65	0.41–1.02	0.06

c) Prevalence at month 18				
Descriptive statistics				
	Sampled (n)	Positive (n)	Positive (%)	
no RAP	391	37	9	
RAP	698	29	4	
**Inferential statistics**				
Model	variable	OR	95% CI	P
unadjusted	RAP	0.38	0.14–0.93	0.03
adjusted	RAP	0.38	0.16–1.00	<0.001
	age 1–3 vs >3 yrs	Na	Na	Na
	age <1 vs >3 yrs	1.28	0.72–2.24	0.14
	sex male	1.16	0.69–1.96	0.11
	FTD-1000 m	1.18	0.85–1.65	0.82
	new animal	0.74	0.4–1.35	0.16

Incidence rate ratios (a) were estimated by Poisson random effect models using individual animal follow up period as offset variable. Odds ratios (b, c) were estimated by logistic random effect models. All models include village as random effect to adjust for correlation within villages. Follow up years; sum of all animals (last month sampled - first month sampled), incidence per 100 years = (Number positive/Follow-up years)*100. CI; confidence interval, OR; Odds ratio.

### Risk of infection with trypanosomes in different regimens

The relative risk of infection with any trypanosome species measured here by the incidence risk ratios was highest in regimen 5 over the 18 months of the study. Cattle in regimen 2 presented with incidence of 5.1 per 100 animal years which was significantly lower compared to the 20.9/100 years observed in the control group (regime 1) (IRR: 0.24; 95% CI: 0.11–0.52; P<0.001) (Table 4). The risk of infection with different trypanosome species was in order of regimen 5>4>3>2 (Table 5). Contrary to our expectation there was no evidence that protection increases with increasing proportion of animals treated.

**Table 4:** Risk of infection with bovine trypanosomes during 18 months follow-up period in different animal treatment regimens (Poisson mixed effect regression model).

Regimen	Number (n)	follow up years	Positive (n)	Incidence per 100 years	IRR	95% CI	P
1	643	674	141	20.9	**Ref**		
2	679	642	33	5.1	0.24	0.11–0.52	<0.001
3	854	813	82	10.1	0.53	0.26–1.08	0.08
4	717	710	96	13.5	0.66	0.33–1.36	0.27
5	562	572	179	31.3	1.57	0.78–3.18	0.21

**Table 5:** Odds ratio of bovine trypanosomes infection after 12 months follow-up in different animal treatment regimens (Logistic mixed effect regression model).

Regimen	Number (n)	Positive; n (%)	OR	95% CI	P
1	404	45 (11%)	**Ref**		
2	285	6 (2%)	0.15	0.04–0.52	0.003
3	426	19 (4%)	0.29	0.10–0.85	0.03
4	353	18 (5%)	0.44	0.16–1.24	0.12
5	339	65 (20%)	1.94	0.73–5.13	0.19

### Apparent tsetse densities in Tororo District; June–September 2012

**Figure 5:**
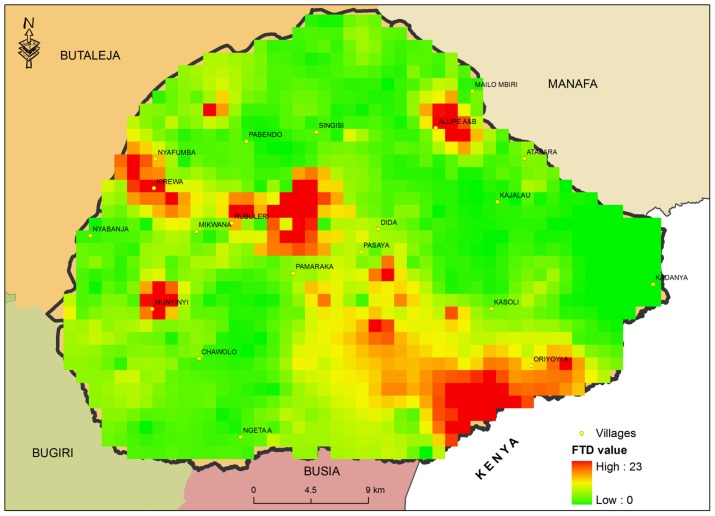
Tororo tsetse apparent density (1 km^2^ grid cell size); June–September 2012.

The number of tsetse flies caught per trap per day were summarised into FTD which was highly variable between traps (Figure 5). About 88% of all tsetse caught during the period were of *G. f. fuscipes* while 12% were *G.pallidipes* from Paya and Mulanda sub counties. *G. pallidipes* was localised at one site in Lwala Parish, Mulanda Sub County but fairly distributed in each of the 4 selected parishes of Paya Sub County (Table 6).

**Table 6:** Apparent tsetse density; Tororo District; June–September 2012.

Sub county	Parishes (n)	Traps (n)	*G. f. fuscipes*			*G. pallidipes*			Sum	Mean FTD (Range)
			M	F	Total	M	F	Total		
Mulanda	3	14	14	13	27	0	1	1	28	0.7 (0–3.3)
Kirewa	5	20	32	28	60	0	0	0	60	1.0 (0–4.0)
Paya	4	15	0	0	0	14	19	33	19	0.6 (0–6.0)
Nagongera	4	14	36	47	83	0	1	1	84	2.0 (0–23.6)
Nabuyoga	4	20	10	5	15	0	1	1	16	0.3 (0–1.6)
Rubongi	5	13	17	21	38	0	0	0	38	1.0 (0–3.6)
Iyolwa	4	18	8	13	21	0	0	0	21	0.4 (0–1.3)
Magola	3	12	15	21	36	0	0	0	36	0.9 (0–1.6)
Osukuru	4	17	59	47	106	0	0	0	106	2.0 (0–6.0)
Kisoko/Petta	5	12	23	20	43	0	0	0	43	1.2 (0–3.3)
Tororo Municipality	2	6	12	4	16	0	0	0	16	1.0 (0–2.6)
**Total**	**43**	**161**	**226**	**219**	**445**	**14**	**22**	**36**	**467**	**1.0 (0–5.2)**

### Association between FTD and baseline trypanosome prevalence

We observed a 2.7%-points increase in the baseline trypanosome prevalence with each 1 unit increase of FTD (95% CI: 0.6–4.7%-points, P = 0.02) using the prediction of the 1 km^2^ spatial aggregation. On a higher spatial aggregation level (25 km^2^ grid cell size) the observed effect was with 1.7%-points smaller and statistically not significant (95% CI: −1.2–4.7%-points, P = 0.26). In Table 7 the baseline prevalences and the corresponding FTD are presented for all villages.

**Table 7:** Baseline trypanosome prevalence and apparent tsetse density.

Village	Trypanosome prevalence	FTD at 1000 M	FTD at 5000 M
Nyafumba	0.18	1.77	1.33
Rubuleri	0.32	1.89	1.65
Singisi	0.26	0.22	0.27
Alupe	0.37	3.26	3.82
Oriyoyi	0.27	1.49	2.39
Mailombiri	0.20	0.08	0.63
Nyabanja	0.31	0.13	0.66
Kajalau	0.28	0.07	0.00
Dida	0.35	0.81	0.23
Ngeta a	0.19	0.18	0.08
Chawolo	0.15	0.17	0.63
Kirewa	0.23	3.28	2.02
Kasoli	0.20	0.54	0.69
Pamaraka	0.19	0.78	0.93
Kadanya	0.20	0.00	0.00
Munyinyi	0.36	4.64	0.88
Pasaya	0.22	0.82	2.26
Atapara	0.21	0.56	0.36
Mikwana	0.21	0.32	1.47
Pabendo	0.29	0.09	0.02

## Discussion

In order to determine the smallest proportion of a village herd that needs to be covered by RAP and effectively control African trypanosomiasis (HAT/AAT), about two thousand cattle in 20 villages of Tororo district were initially introduced into the 18 months RAP optimisation trial. Over the period, about 1700 cattle were introduced into the trial. Cattle in all the 20 study villages were predominantly hybrids of the African short horn Zebu (Nkedi) and Boran breed. The Nkedi cattle and their hybrids with Boran are the most predominant cattle breeds in this region [33], [42]. At the beginning of the trial, 46% of the cattle were above 3 years of age with 45.6% of all cattle being either whole or neutered males. This population structure of retaining more old cattle with a female to male (whole and neutered males) ratio of nearly 1 is geared towards creating a mass of draught power animals [33], [42]. Cattle above three years of age have recently been seen to be associated with higher risk of infection with and spread of human infective *T.b rhodesiense* [43].These production systems that retain a very high proportion of cattle above 3 years of age pose a higher risk of acute HAT transmission. This implies that improving livestock health by controlling tsetse and trypanosomiasis will ultimately block zoonotic trypanosomiasis transmission. In addition, this will pave way to integrating livestock and crop production by way of using draught power and cattle manure in crop production. This is in line with previous recommendations that controlling trypanosomiasis in small holder crop-livestock production systems where farmers heavily depend on draught power and cattle manure in their crop production will help reduce poverty and hunger [1], [2], [20], [24].

As with previous studies [20]–[22] this study shows that restricting pyrethroid insecticides to the legs, bellies and ears of cattle significantly reduces trypanosome infections in sprayed cattle compared to unsprayed cattle. RAP was particularly effective at preventing re-infection with *T. brucei s.l.* indicating that it could be effective at controlling acute HAT since cattle are known reservoirs for *T. brucei rhodesiense* [6]–[9].

Despite the fact that RAP was generally effective at preventing re-infections with different trypanosome species, increase in village RAP herd coverage was not significantly associated with a proportionate decrease in the trypanosome prevalence. On the contrary, there was an inverse relationship between dose (increase in RAP coverage; 25%RAP>50%RAP>75%RAP) and response (reduction in trypanosome prevalence). Inter-village distances and drop-in effect (cattle introductions) were not significant predictors of infection with trypanosome infections. However, herd (village) level trypanosome prevalence varied greatly in different parts of Tororo district with the highest in central and north-western parts of the district during intervention (Figure 4). This indicates that there were village level effects (p; 0.02) that caused this observation.

As previously reported, the majority (88%) of the tsetse caught were *G. f. fuscipes* [9] while the rest were *G. pallidipes* distributed in rather patchy areas in Mulanda and Paya sub counties. *G.pallidipes* has previously [31] been linked to re-introductions from Busia District, Kenya further confirming its patchy distribution and small numbers in this study. There was some indication that FTD-1000 m had an impact on the treatment effect, but the association was only statistically significant in one of the 3 models. Consistent with literature [44], FTD alone did not sufficiently explain trypanosome infection rates at the beginning of the trial. This would imply that the observed village level effect in trypanosome prevalence during the trial (Figures 4 and 5) that caused the rather unexpected distortion in dose-response relationship was multifactorial. Such factors that could explain this include a product of FTD and mean tsetse fly infection rate described elsewhere as level of challenge [44]. Other factors include differences in individual tsetse-trypanosome transmission rates and cattle management practices between villages.

The observations above are important in re-focusing the way insecticide treated cattle (ITC); RAP in particular, is used in the control of African trypanosomiasis. Recent mathematical models, for example, predict that just as low as 20–27% RAP coverage is sufficient in controlling *T. brucei s.l.* [45], [46]. The current study is consistent with this prediction. In fact *T. brucei s.l.* was detected in negligible proportions at all sampling points after DA treatments in all the RAP villages compared to the non-RAP villages.

The last outbreak of sleeping sickness in Tororo was between 1988–1990 [47]. This was followed by government, livestock and donor-led control programs that resulted in the control of the disease in humans and probably a significant reduction in the animal reservoir [48]. For the last 20 years *T. brucei s.l* cattle reservoir seems to have remained low (3–7%) a reason as to why there have not been recent outbreaks of sleeping sickness in Tororo district [6], [49]. It is particularly for this reason that we chose to optimise RAP in this region where trypanosome (especially *T. brucei s.l* group) transmission dynamics are quite in a stable state. As well, there have not been massive cattle restocking in Tororo district like there have been in the Teso sub region. Cattle restocking has been reported as the major reason why *T. brucei s.l* particularly *T. b. rhodesiense* has been on the increase in this region resulting into the recent sleeping sickness outbreak in the region and its northerly spread towards the chronic HAT focus [15], [18], [50]. This implies that treatment of 25% of a village herd in a stable disease transmission state where cattle are the main reservoir for *T. brucei s.l* is sufficient for the control of this group of trypanosomes with likely long lasting effects on the control of acute sleeping sickness.

On the other hand *T. vivax* and *T. c. savannah* were detected in much higher proportions in both RAP and non-RAP villages at all sampling points after treatment with DA. Consistent with literature [45], *T. vivax* was the most persistent trypanosome species detected in both RAP and non-RAP villages during the 18 months trial. This could have been caused by mechanical transmission by several populations of biting flies whose homing and feeding sites might not be those preferred by tsetse (legs, bellies) [51], [52]. Similarly, it could have been as a result of higher challenge of *T.vivax* and *T.congolense* since these trypanosome species were detected in much higher proportions 9 months before intervention. This implies that control of nagana (*T.vivax* and *T.congolense*) in areas where tsetse mainly feed on cattle would require increasing village RAP herd coverage up to 50–75% as previously suggested [45]. In the current situation where dose response is distorted probably due to the differences in village level challenge, trypanosome transmission rates and management practices, initial treatment of all cattle in the intervention area with a curative trypanocide to reduce parasitaemia would leverage control by denying tsetse of trypanosomes to transmit [46]. This could be repeated once yearly for the first three or so years in the control program in moderate to high tsetse density areas. As such, the fact that we did not treat all cattle in Tororo district with Veriben B12 at the beginning of this trial could have contributed to the observed distortion in the dose-response relationship. Similarly, if RAP were adopted across all villages, this would have significantly increased the effectiveness of RAP and further reduced the distortion in the dose-response relationship. There is therefore need to do this assessment in future studies.

### Conclusion

This study complements the available literature to demonstrate that RAP is effective in controlling African trypanosomiasis. To our knowledge, this study provides the first field based longitudinal study to demonstrate that spraying only as low as 25% of a village cattle herd in stable African trypanosomiasis transmission area is sufficient in the control of T. *brucei s.l.* In high tsetse challenge areas where tsetse mainly feed on cattle, control of nagana (*T.vivax* and *T.congolense*) would probably require increasing village RAP herd coverage to 50–75% without reducing RAP efficacy. This is particularly important because *T. vivax* and *T. c. savannah* persist under moderate (<50% RAP) tsetse control over a long period of time. In such areas, treatment of all cattle with a curative trypanocide once yearly for the first 1–2 years of the control program would leverage tsetse control by reducing parasitaemia. Reducing RAP coverage to 25% (T. *brucei s.l* control) or 50–75% (*T.vivax*/*congolense* control) would further reduce the amount of insecticides used compared to that used in whole body spraying. This will further reduce cost of application of RAP and improve uptake by small holder farmers in many crop-livestock production systems. Before these findings are integrated in routine tsetse control programs we recommend that the performance of different RAP herd coverage levels is evaluated in varied tsetse challenge, trypanosome transmission rates and management systems.

## Supporting Information

Supporting Information S1 **a**) **Impact of RAP on trypanosome prevalence at each follow-up sampling point.** Logistic regression with village level random effect to account for correlation within herds. In the adjusted models the effect of RAP is adjusted for the covariates: age category, sex, tsetse density at baseline (predicted from spatial extrapolation) and if an animal received treatment at baseline (Veriben B12- vs. drop-in during follow-up). **b**) **Impact of RAP on trypanosome prevalence.** Logistic regression with village level random effect to account for correlation within herds and treatment×time interaction as outcome. Reference time point is 14 days after the Veriben B12 injections at baseline. Presented coefficients are on logit scale.
(DOCX)
