# Supplementary material for: Improvements on Restricted Insecticide Application Protocol for Control of Human and Animal African Trypanosomiasis in Eastern Uganda
Source: PLoS Negl Trop Dis. 2014 Oct 30;8(10):e3284. doi: 10.1371/journal.pntd.0003284 (PMC4214683; doi:10.1371/journal.pntd.0003284)
Supplement: Supporting Information S1 — a) Impact of RAP on trypanosome prevalence at each follow-up sampling point. Logistic regression with village level random effect to account for correlation within herds. In the adjusted models the effect of RAP is adjusted for the covariates: age category, sex, tsetse density at baseline (predicted from spatial extrapolation) and if an animal received treatment at baseline (Veriben B12- vs. drop-in during follow-up). b) Impact of RAP on trypanosome prevalence. Logistic regression with village level random effect to account for correlation within herds and treatment×time interaction as outcome. Reference time point is 14 days after the Veriben B12 injections at baseline. Presented coefficients are on logit scale. (DOCX) [file pntd.0003284.s001.docx]

Supplementary analyses – A cluster randomized trial to quantify the effect of restricted pyrethroid insecticide application to the legs, belly and ears (RAP) for trypanosomiasis control in cattle.

1. **Impact of RAP on trypanosome prevalence at each follow-up sampling point.**

| Month | Model | Number (n) | Odds ratio (OR) | 95% CI | P |
| --- | --- | --- | --- | --- | --- |
| 3 | Unadjusted | 1692 | 0.45 | 0.19 - 0.99 | 0.04 |
|  | Adjusted | 1692 | 0.46 | 0.19 - 1.01 | 0.05 |
| 6 | Unadjusted | 1899 | 0.18 | 0.05 - 0.53 | 0.002 |
|  | Adjusted | 1899 | 0.18 | 0.05 - 0.53 | 0.002 |
| 9 | Unadjusted | 1665 | 0.40 | 0.16 - 0.93 | 0.03 |
|  | Adjusted | 1665 | 0.43 | 0.18 - 0.97 | 0.04 |
| 12 | Unadjusted | 1965 | 0.20 | 0.08 - 0.44 | <0.0001 |
|  | Adjusted | 1965 | 0.22 | 0.10 - 0.48 | 0.0001 |
| 15 | Unadjusted | 1134 | 0.25 | 0.08 - 0.79 | 0.01 |
|  | Adjusted | 1134 | 0.27 | 0.08 - 0.87 | 0.02 |
| 18 | Unadjusted | 1089 | 0.38 | 0.14 - 0.93 | 0.03 |
|  | Adjusted | 1089 | 0.42 | 0.16 - 1.01 | 0.04 |

1. **Impact of RAP on trypanosome prevalence.**

| Covariate | coefficient | SE | Z | P |
| --- | --- | --- | --- | --- |
| *Intercept* | *-4.28* | *0.39* | *-10.96* | *<0.0001* |
| Month 3 | 1.38 | 0.34 | 4.05 | <0.0001 |
| Month 6 | 2.18 | 0.32 | 6.91 | <0.0001 |
| Month 9 | 2.04 | 0.32 | 6.39 | <0.0001 |
| Month 12 | 2.45 | 0.31 | 7.88 | <0.0001 |
| Month 15 | 2.71 | 0.32 | 8.36 | <0.0001 |
| Month 18 | 1.88 | 0.34 | 5.54 | <0.0001 |
| RAP × month 0 | -0.43 | 0.52 | -0.83 | 0.40 |
| RAP × month 3 | -0.82 | 0.42 | -1.93 | 0.05 |
| RAP × month 6 | -1.51 | 0.40 | -3.77 | 0.0002 |
| RAP × month 9 | -0.89 | 0.39 | -2.28 | 0.02 |
| RAP × month 12 | -1.53 | 0.39 | -3.95 | <0.0001 |
| RAP × month 15 | -1.58 | 0.40 | -3.95 | <0.0001 |
| RAP × month 18 | -0.98 | 0.43 | -2.31 | 0.02 |
